# Supplementary material for: Internal states as a source of subject-dependent movement variability are represented by large-scale brain networks
Source: Nat Commun. 2023 Nov 29;14:7837. doi: 10.1038/s41467-023-43257-4 (PMC10687170; doi:10.1038/s41467-023-43257-4)
Supplement: Supplementary file 1 — Supplementary Information [file 41467_2023_43257_MOESM1_ESM.pdf]

Internal states as a source of subject-dependent movement variability are represented by large-scale brain networks

Macauley Smith Breault, Pierre Sacré, Zachary B. Fitzgerald, John T. Gale, Kathleen E. Cullen, Jorge A. González-Martínez, Sridevi V. Sarma

Supplementary Information

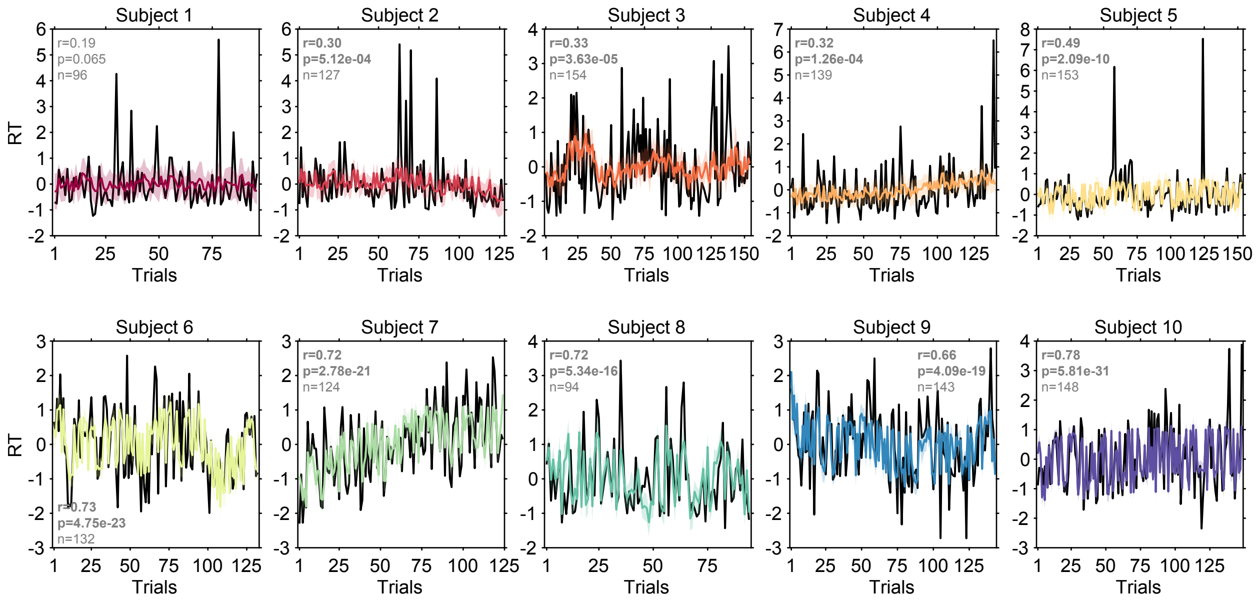

Supplementary Figure 1 | Estimation of reaction time (RT). Overlay of observed RT (black solid line) and estimated RT (solid color line) across all trials. The RT of each subject was normalized using the z-score before fitting and plotting the models. Each subject is colored in their panel. The shaded color represents the 95 % confidence interval of the estimated RT. Inset are the statistical results of two-tailed Pearson correlation between observed and estimated RT, which are bold if significant. Source data are provided as a Source Data file.

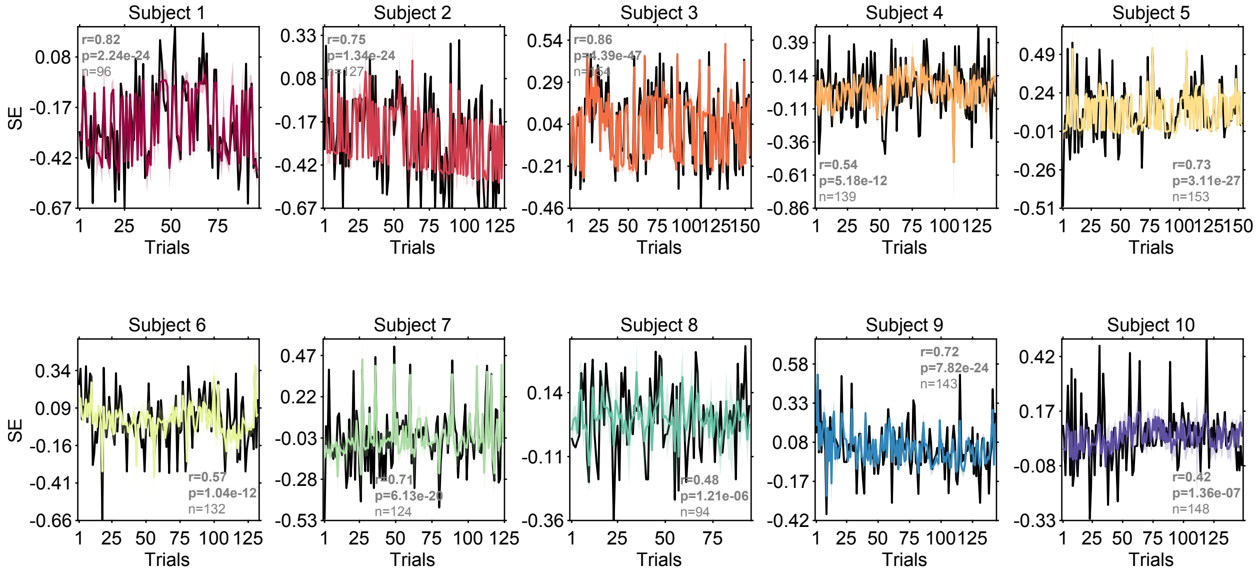

Supplementary Figure 2 | **Estimation of speed error (SE).** Overlay of observed SE (black solid line) and estimated SE (solid color line) across all trials. Each subject is colored in their panel. The shaded color represents the 95 % confidence interval of the estimated SE. Inset are the statistical results of two-tailed Pearson correlation between observed and estimated SE, which are bold if significant. Source data are provided as a Source Data file.

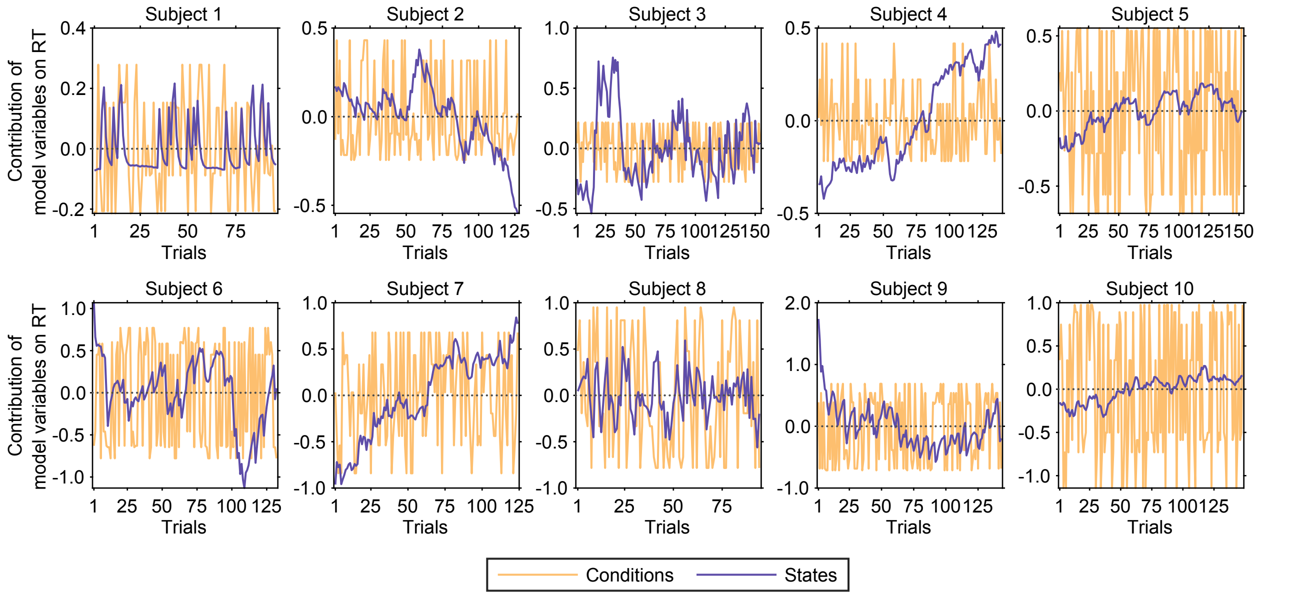

Supplementary Figure 3 | **Contribution of model variables to reaction time (RT).** Contribution of the internal states (purple solid line) and trial conditions (orange solid line) on the estimated RT across trials. The origin is marked by the horizontal dark grey dotted line. Source data are provided as a Source Data file.

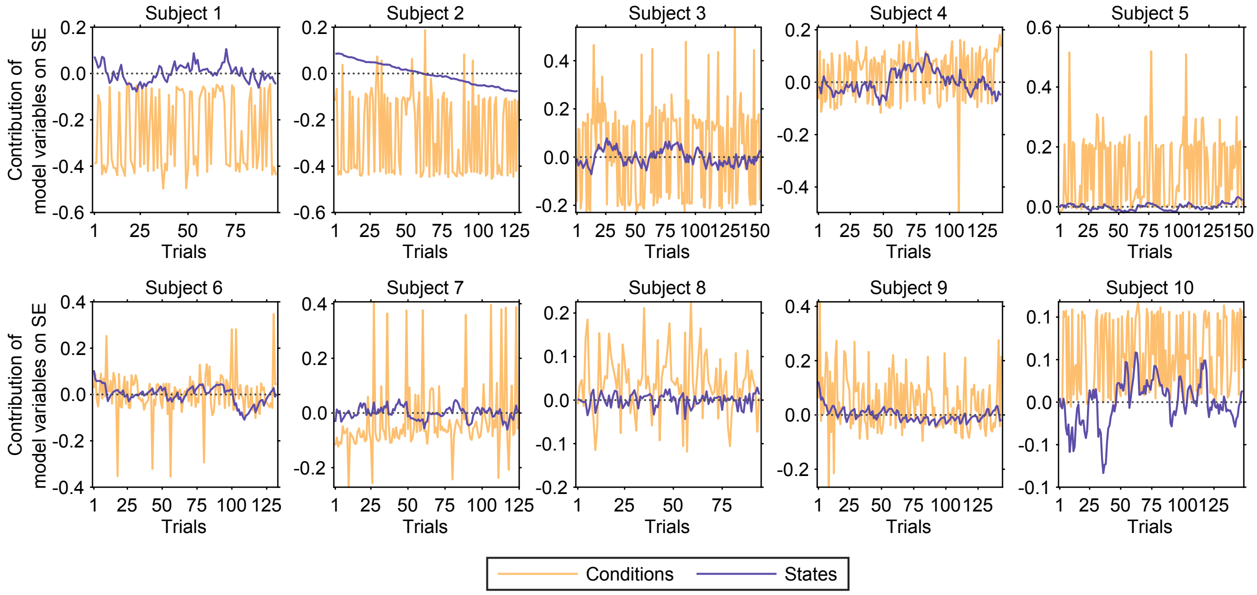

Supplementary Figure 4 | Contribution of model variables to speed error (SE). Contribution of the internal states (purple solid line) and trial conditions (orange solid line) on the estimated SE across trials. The origin is marked by the horizontal dark grey dotted line. Source data are provided as a Source Data file.

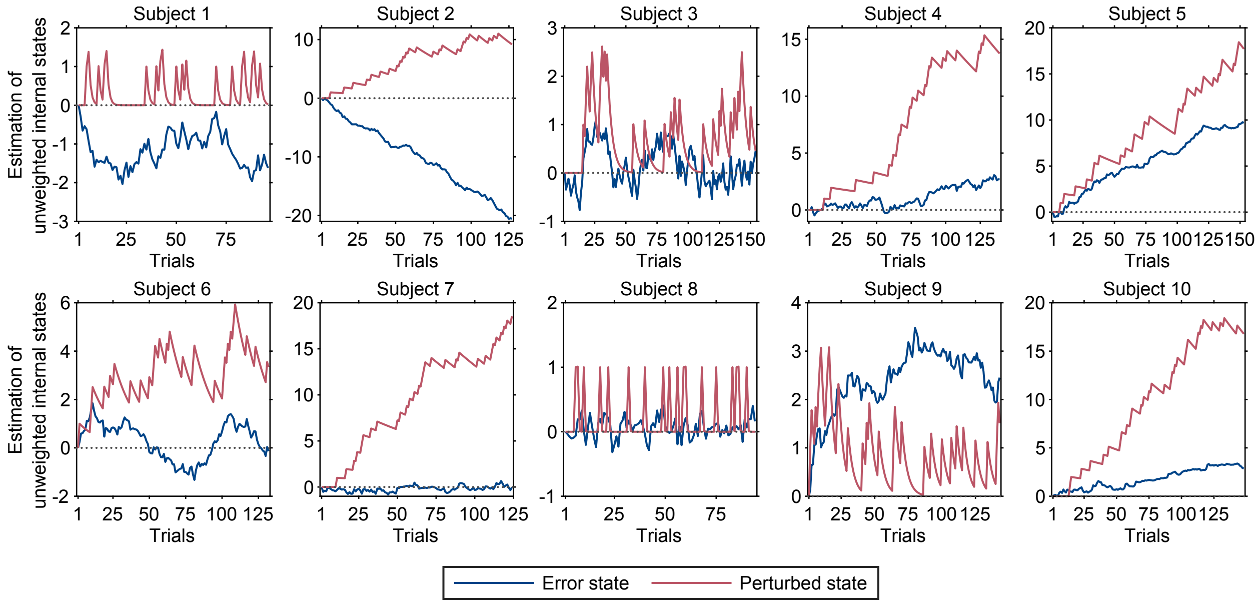

Supplementary Figure 5 | Estimation of internal states. Estimation of error state (blue solid line) and perturbed state (pink solid line) over trials for each subject. The states were used to estimate both reaction time (RT) and speed error (SE) by fitting each model with weights that optimized the combined model performance. The y-axis here does not reflect the actual values used in equations (1) and (2) because the internal states were normalized using the z-score before fitting. A state of 0 is marked by the horizontal dark grey dotted line. Source data are provided as a Source Data file.

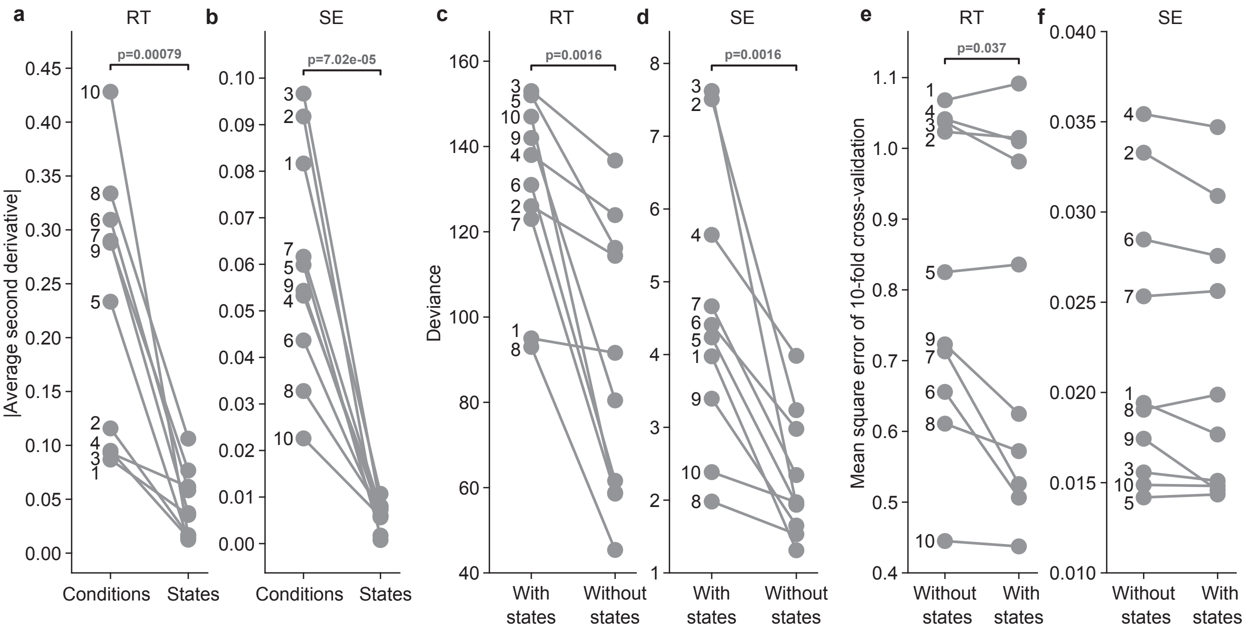

Supplementary Figure 6 | **Performance of behavioral model.** **a** Absolute average second derivative of the conditions and states for the (**a**) reaction time (RT) and (**b**) speed error (SE) models across all subjects. We found a significant difference between conditions and states for RT (two-tailed paired-sample t-test: $t(9) = 4.96$, $p = 0.0008$) and SE (two-tailed paired-sample t-test: $t(9) = 6.91$, $p = 7.02\times^{-5}$). This shows that conditions exhibited sudden changes between trials while states exhibited gradual changes across all subjects. Deviance for the (**c**) RT and (**d**) SE models across all subjects. We compared the behavioral models with internal states (With states) to a model with the same trial conditions but without internal states (Without states). Each marker is labeled by the subject it represents. Adding internal states significantly improved the goodness-of-fit of the RT (two-tailed paired-sample t-test: $t(9) = 4.46$, $p = 0.0016$) and SE (two-tailed paired-sample t-test: $t(9) = 4.45$, $p = 0.0016$) model for all subjects, as larger values are better. 10-fold cross-correlation performance, measured using mean square error, for the (**e**) RT and (**f**) SE models across all subjects. We found that adding internal states significantly improved the model's ability to predict RTs (two-tailed paired-sample t-test: $t(9) = 2.45$, $p = 0.037$) but not SE. Source data are provided as a Source Data file.

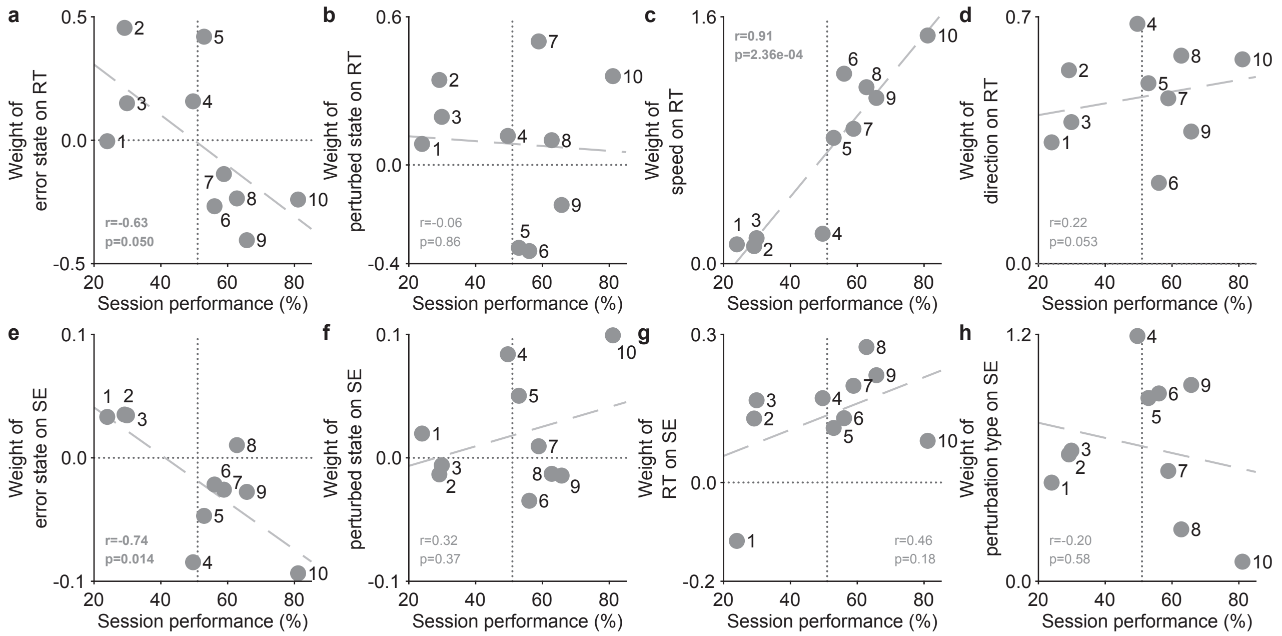

Supplementary Figure 7 | Comparison between model weights and session performance. Markers are colored for each subject. Weight of (**a**) error state, (**b**) perturbed state, (**c**) 2-norm of speeds, and (**d**) 2-norm of directions on reaction time (RT) from equation (1) over session performance. Weight of (**e**) error state, (**f**) perturbed state, (**g**) RT, and (**h**) 2-norm of perturbation types on speed error (SE) from equation (2) over session performance. Average session performance (51 %) is marked by the vertical dark grey dotted line. A weight of 0 is marked by the horizontal dark grey dotted line. The least-squares line is marked as the light grey dashed line. Inset are the statistical results of the two-tailed Pearson correlation between weight and session performance, which are bold if significant. Source data are provided as a Source Data file.

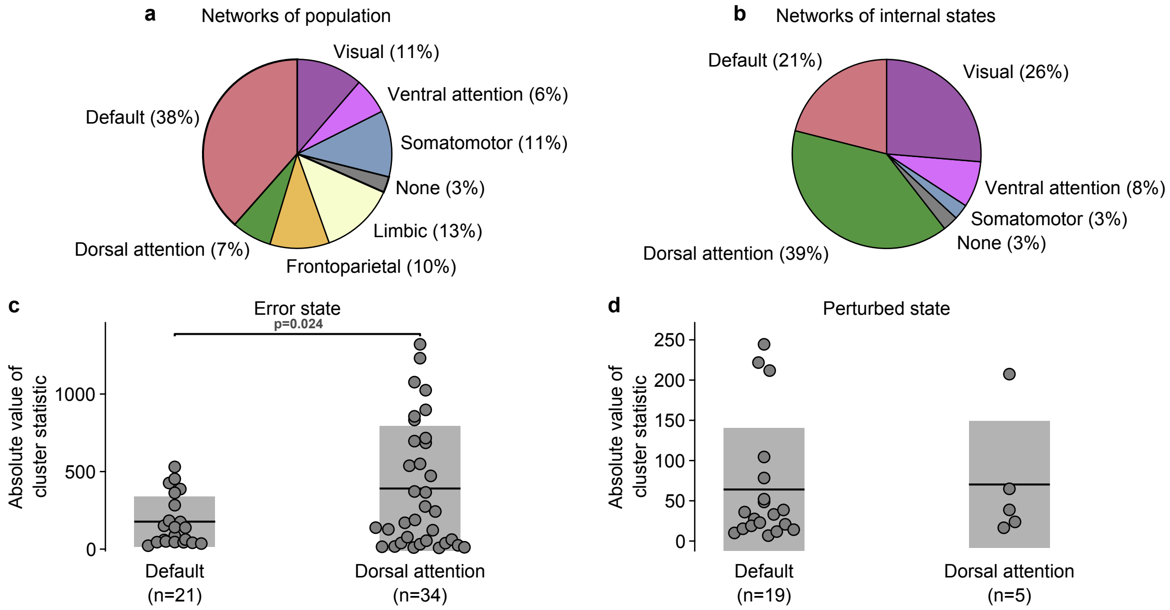

Supplementary Figure 8 | Summary of cluster statistic on large-scale brain networks. Coverage of electrodes across large-scale brain networks in the population (**a**) before and (**b**) after filtering those involved with the internal states and performance from the nonparametric cluster statistic. Comparing the absolute value of the cluster statistic from regions in the default network (DN) and dorsal attention network (DAN) for the (**c**) error state and (**d**) perturbed state using a two-tailed two-sample t-test. The higher the magnitude of the cluster statistic, the greater the statistical significance of the cluster is. We found a significant difference between DN and DAN for the error state (two-tailed two-sample t-test: $t(53) = 2.32, p = 0.024$) but not the perturbed state (two-tailed two-sample t-test: $t(22) = 0.16, p = 0.87$). Source data are provided as a Source Data file.

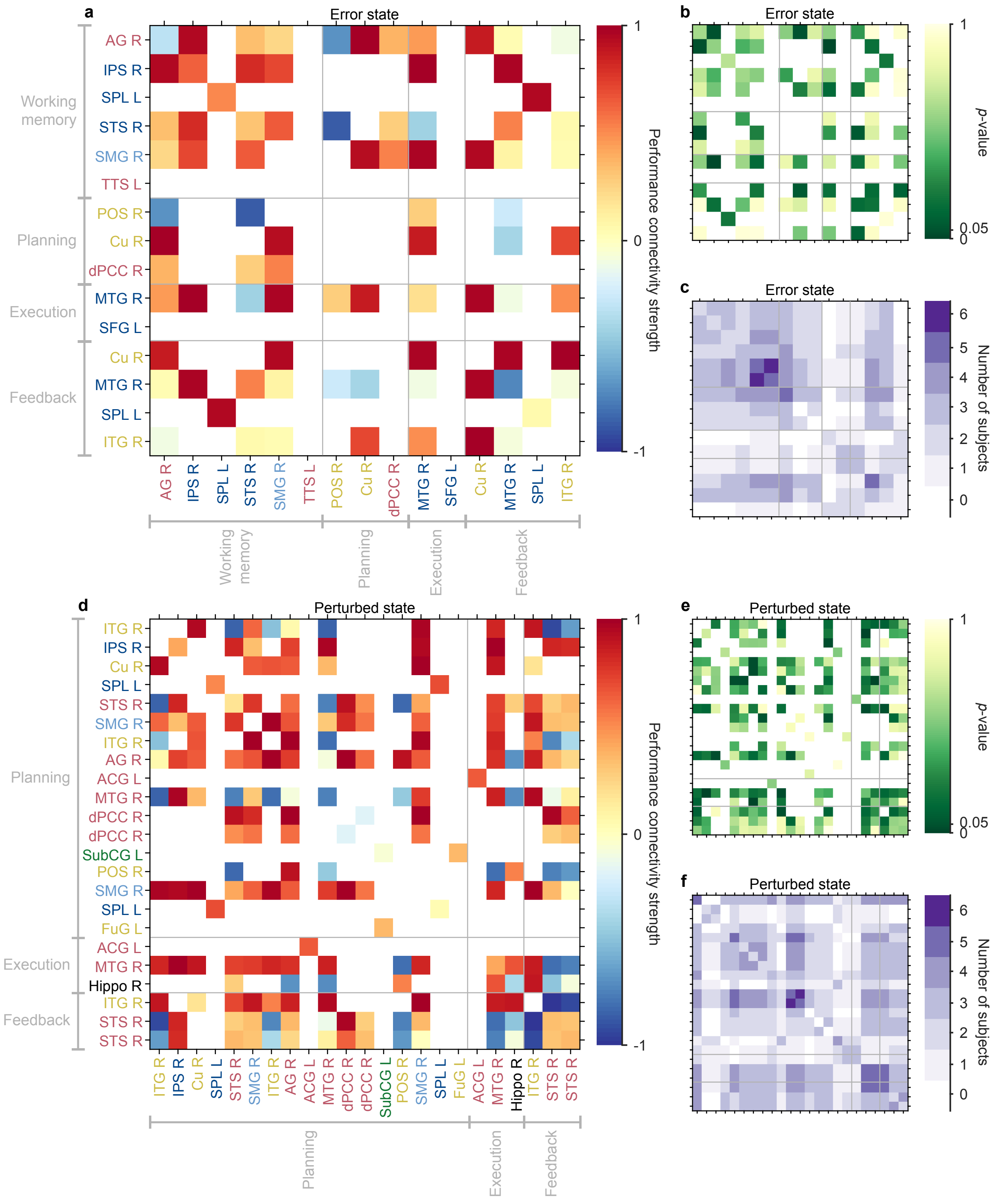

Supplementary Figure 9 | Performance connectivity strength. Performance connectivity strength as a heat map for the (**a**) error state and (**d**) perturbed state. Labels match abbreviation using the order from Table 2 and Table 3. They are colored by the network they belong too: dorsal attention network (DAN) in dark blue, default network (DN) in red, ventral attention network (VAN) in light blue, visual in yellow, somatomotor in green, none in black. The relationship between performance and subject connectivity strengths is represented by the sign and magnitude of two-tailed Pearson correlation value and takes on a value between -1 and 1, where -1 (blue) being a negative correlation, 0 (yellow) being no relationship and 1 (red) being a positive correlation. If connectivity could not be calculated, then the pixel is colored white. The p-values (**b** and **e**) and number of subjects (**c** and **f**) used to calculate this connectivity are also shown using the same label order. Source data are provided as a Source Data file.

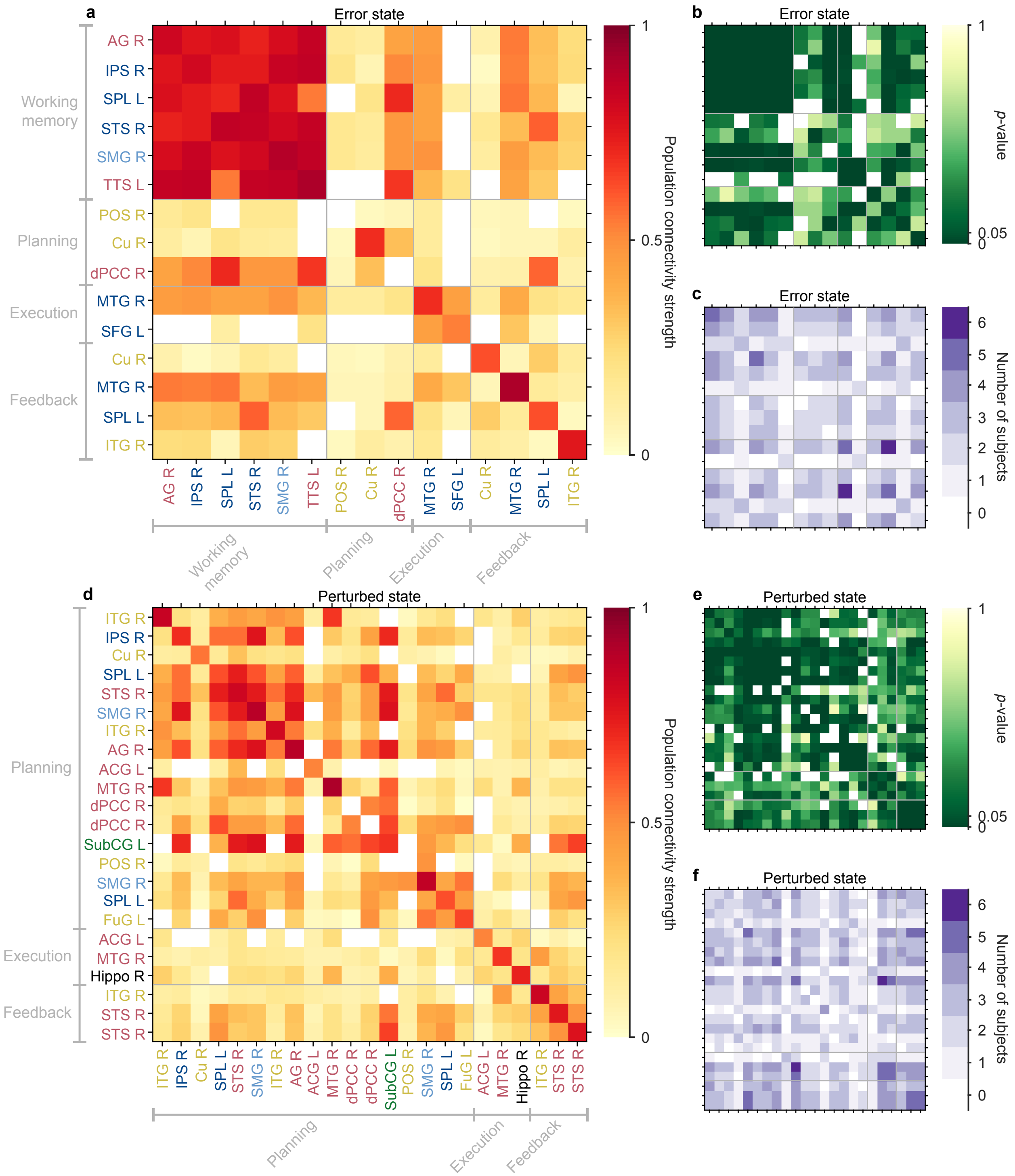

Supplementary Figure 10 | Population connectivity strength. Population connectivity strength as a heat map for the (**a**) error state and (**d**) perturbed state. Labels match abbreviation using the order from Table 2 and Table 3. They are colored by the network they belong to: dorsal attention network (DAN) in dark blue, default network (DN) in red, ventral attention network (VAN) in light blue, visual in yellow, somatomotor in green, none in black. Connectivity strengths are the average magnitude of the subject connectivity values between 0 and 1, with 0 (yellow) being no relationship and 1 (red) being completely in sync. If connectivity could not be calculated, then the pixel is colored white. The average p-values (**b** and **e**) and number of subjects (**c** and **f**) used to calculate this connectivity are also shown using the same label order. Source data are provided as a Source Data file.

Subject	Reaction time (s)	Speed error
1	0.98 ± 0.26	-0.26 ± 0.20
2	0.80 ± 0.18	-0.26 ± 0.24
3	0.91 ± 0.19	0.03 ± 0.22
4	0.93 ± 0.22	0.03 ± 0.20
5	0.66 ± 0.20	0.12 ± 0.17
6	1.07 ± 0.27	0.00 ± 0.18
7	0.73 ± 0.12	-0.03 ± 0.19
8	0.80 ± 0.27	0.04 ± 0.15
9	0.67 ± 0.18	0.04 ± 0.15
10	0.52 ± 0.11	0.05 ± 0.13

Supplementary Table 1 | Summary (mean ± 1 standard deviation) of reaction time and speed error for each subject across all completed trials.

Subject	Reaction time (s)						Speed error					
	Speed		Direction				Perturbation type					
	fast	slow	down	right	up	left	fast, unpert.	slow, unpert.	fast, towards	slow, towards	fast, away	slow, away
1	0.99 ± 0.32	0.97 ± 0.19	0.94 ± 0.16	0.94 ± 0.30	1.03 ± 0.19	0.99 ± 0.37	-0.08 ± 0.14	-0.40 ± 0.12	-0.11 ± 0.18	-0.37 ± 0.07	-0.07 ± 0.21	-0.44 ± 0.11
2	0.81 ± 0.20	0.79 ± 0.17	0.79 ± 0.20	0.77 ± 0.10	0.87 ± 0.21	0.77 ± 0.18	-0.10 ± 0.15	-0.43 ± 0.17	-0.18 ± 0.17	-0.39 ± 0.31	0.09 ± 0.22	-0.14 ± 0.20
3	0.92 ± 0.20	0.90 ± 0.17	0.87 ± 0.16	0.90 ± 0.17	0.93 ± 0.23	0.94 ± 0.19	0.15 ± 0.14	-0.19 ± 0.10	0.32 ± 0.19	-0.04 ± 0.20	0.43 ± 0.04	0.05 ± 0.11
4	0.91 ± 0.18	0.95 ± 0.27	0.89 ± 0.19	0.94 ± 0.20	0.99 ± 0.20	0.92 ± 0.29	0.10 ± 0.19	-0.07 ± 0.17	0.13 ± 0.28	-0.51 ± 0.00	0.12 ± 0.12	0.05 ± 0.09
5	0.57 ± 0.09	0.74 ± 0.22	0.60 ± 0.12	0.64 ± 0.11	0.71 ± 0.32	0.71 ± 0.12	0.21 ± 0.12	0.01 ± 0.10	0.52 ± 0.05	0.14 ± 0.06	0.30 ± 0.21	0.06 ± 0.09
6	0.90 ± 0.23	1.23 ± 0.21	1.05 ± 0.23	1.06 ± 0.36	1.07 ± 0.24	1.11 ± 0.26	0.05 ± 0.19	-0.04 ± 0.10	0.00 ± 0.32	-0.32 ± 0.24	0.30 ± 0.05	0.02 ± 0.10
7	0.66 ± 0.10	0.78 ± 0.11	0.77 ± 0.11	0.67 ± 0.14	0.73 ± 0.10	0.72 ± 0.12	-0.09 ± 0.16	-0.05 ± 0.12	-0.10 ± 0.41	-0.25 ± 0.22	0.39 ± 0.09	0.09 ± 0.05
8	0.66 ± 0.13	0.99 ± 0.28	0.76 ± 0.25	0.69 ± 0.19	0.89 ± 0.34	0.89 ± 0.25	0.05 ± 0.16	0.01 ± 0.13	-0.07 ± 0.21	0.02 ± 0.05	0.16 ± 0.10	0.05 ± 0.11
9	0.56 ± 0.16	0.75 ± 0.13	0.66 ± 0.15	0.69 ± 0.20	0.69 ± 0.16	0.63 ± 0.20	0.09 ± 0.12	-0.04 ± 0.10	0.51 ± 0.00	-0.22 ± 0.22	0.24 ± 0.17	0.05 ± 0.12
10	0.44 ± 0.06	0.60 ± 0.09	0.52 ± 0.10	0.55 ± 0.12	0.48 ± 0.08	0.54 ± 0.13	0.11 ± 0.16	0.01 ± 0.10	0.05 ± 0.05	0.01 ± 0.00	0.06 ± 0.09	0.03 ± 0.07

Supplementary Table 2 | Summary (mean ± 1 standard deviation) of reaction time and speed error for each subject across all completed trials based on trial conditions (*e.g.*, speed, direction, and perturbation type).

Subject	Completed	Number of trials									Session performance (%)
		Speed		Direction				Perturbation			
		fast	slow	down	right	up	left	unpert.	towards	away	
1	96	45	51	27	20	26	23	76	8	12	24
2	127	53	74	43	39	34	30	102	10	15	29
3	154	81	73	37	38	43	36	128	6	20	30
4	139	77	62	39	36	29	35	115	8	16	50
5	153	68	85	36	45	39	33	122	7	24	53
6	132	64	68	37	30	33	32	105	13	14	56
7	124	56	68	38	30	34	22	94	8	22	59
8	94	53	41	16	29	20	29	76	6	12	63
9	143	65	78	46	29	36	32	113	3	27	66
10	148	74	74	31	43	39	35	117	7	24	81

Supplementary Table 3 | Number of completed trials for each trial conditions (*e.g.*, speed, direction, perturbation) and session performance for each subject.

Factor	df	SS	MS	EMS	F	p-value
subject	9	34.53	3.84	126.796*V(subject)+63.3982*V(subject*speed)+31.6991*V(subject*direction)+V(Error)	7.24	2.85×10^{-03}
speed	1	4.87	4.87	615.336*Q(speed)+61.5336*V(subject*speed)+153.834*Q(speed*direction)+V(Error)	9.74	1.22×10^{-02}
direction	3	0.66	0.22	308.544*Q(direction)+30.8544*V(subject*direction)+154.272*Q(speed*direction)+V(Error)	4.59	9.66×10^{-03}
subject*speed	9	4.63	0.51	63.3367*V(subject*speed)+V(Error)	15.65	1.88×10^{-24}
subject*direction	27	1.30	0.05	32.1303*V(subject*direction)+V(Error)	1.47	5.78×10^{-02}
speed*direction	3	0.18	0.06	158.16*Q(speed*direction)+V(Error)	1.85	1.37×10^{-01}
Error	1257	41.29	0.03	V(Error)		
Total	1309	87.50				

Supplementary Table 4 | Group data analysis using a three-way ANOVA of main effects and interactions that subject, speed, and direction have on reaction time. (df = degrees of freedom, SS = Sum of Squares, MS = Mean Squares, EMS = Expected Mean Squares, F = F-statistic)

Factor A	Factor B	Lower Limit	Mean Difference	Upper Limit	p-value
subject=1	subject=2	0.10	0.18	0.26	0.00
subject=1	subject=3	-0.01	0.07	0.14	0.12
subject=1	subject=4	-0.03	0.05	0.12	0.67
subject=1	subject=5	0.25	0.32	0.40	0.00
subject=1	subject=6	-0.17	-0.09	-0.01	0.01
subject=1	subject=7	0.18	0.26	0.34	0.00
subject=1	subject=8	0.07	0.15	0.24	0.00
subject=1	subject=9	0.24	0.32	0.40	0.00
subject=1	subject=10	0.38	0.45	0.53	0.00
subject=2	subject=3	-0.18	-0.11	-0.04	0.00
subject=2	subject=4	-0.20	-0.13	-0.06	0.00
subject=2	subject=5	0.07	0.14	0.21	0.00
subject=2	subject=6	-0.34	-0.27	-0.20	0.00
subject=2	subject=7	0.00	0.08	0.15	0.03
subject=2	subject=8	-0.11	-0.03	0.05	0.99
subject=2	subject=9	0.07	0.14	0.21	0.00
subject=2	subject=10	0.20	0.28	0.35	0.00
subject=3	subject=4	-0.09	-0.02	0.05	0.99
subject=3	subject=5	0.19	0.26	0.32	0.00
subject=3	subject=6	-0.23	-0.16	-0.09	0.00
subject=3	subject=7	0.12	0.19	0.26	0.00
subject=3	subject=8	0.01	0.09	0.16	0.02
subject=3	subject=9	0.19	0.25	0.32	0.00
subject=3	subject=10	0.32	0.39	0.45	0.00
subject=4	subject=5	0.21	0.28	0.35	0.00
subject=4	subject=6	-0.21	-0.14	-0.07	0.00
subject=4	subject=7	0.14	0.21	0.28	0.00
subject=4	subject=8	0.03	0.11	0.19	0.00
subject=4	subject=9	0.20	0.27	0.34	0.00
subject=4	subject=10	0.34	0.41	0.48	0.00
subject=5	subject=6	-0.48	-0.41	-0.35	0.00
subject=5	subject=7	-0.14	-0.07	0.01	0.10
subject=5	subject=8	-0.25	-0.17	-0.09	0.00
subject=5	subject=9	-0.07	-0.00	0.07	1.00
subject=5	subject=10	0.06	0.13	0.20	0.00
subject=6	subject=7	0.28	0.35	0.42	0.00
subject=6	subject=8	0.17	0.24	0.32	0.00
subject=6	subject=9	0.34	0.41	0.48	0.00
subject=6	subject=10	0.48	0.55	0.61	0.00
subject=7	subject=8	-0.19	-0.10	-0.02	0.00
subject=7	subject=9	-0.01	0.06	0.13	0.15
subject=7	subject=10	0.13	0.20	0.27	0.00
subject=8	subject=9	0.09	0.17	0.25	0.00
subject=8	subject=10	0.22	0.30	0.38	0.00
subject=9	subject=10	0.07	0.13	0.20	0.00

Supplementary Table 5 | Group data analysis using a post-hoc Tukey's comparison for the main effect of subject on reaction time.

Factor A	Factor B	Lower Limit	Mean Difference	Upper Limit	p-value
direction=down	direction=left	-0.07	-0.03	0.00	0.09
direction=down	direction=right	-0.04	-0.01	0.03	0.99
direction=down	direction=up	-0.09	-0.06	-0.02	0.00
direction=left	direction=right	-0.01	0.03	0.07	0.20
direction=left	direction=up	-0.06	-0.02	0.02	0.42
direction=right	direction=up	-0.09	-0.05	-0.01	0.00

Supplementary Table 6 | Group data analysis using a post-hoc Tukey's comparison for the main effect of direction on reaction time.

Factor A	Factor B	Lower Limit	Mean Difference	Upper Limit	p-value
speed=fast,direction=down	speed=slow,direction=down	−0.15	−0.09	−0.03	0.00
speed=fast,direction=down	speed=fast,direction=left	−0.06	−0.00	0.06	1.00
speed=fast,direction=down	speed=slow,direction=left	−0.22	−0.16	−0.10	0.00
speed=fast,direction=down	speed=fast,direction=right	−0.05	0.01	0.07	1.00
speed=fast,direction=down	speed=slow,direction=right	−0.18	−0.11	−0.05	0.00
speed=fast,direction=down	speed=fast,direction=up	−0.10	−0.04	0.02	0.52
speed=fast,direction=down	speed=slow,direction=up	−0.23	−0.17	−0.10	0.00
speed=slow,direction=down	speed=fast,direction=left	0.03	0.09	0.16	0.00
speed=slow,direction=down	speed=slow,direction=left	−0.13	−0.07	−0.01	0.02
speed=slow,direction=down	speed=fast,direction=right	0.04	0.10	0.16	0.00
speed=slow,direction=down	speed=slow,direction=right	−0.08	−0.02	0.04	0.97
speed=slow,direction=down	speed=fast,direction=up	−0.01	0.05	0.11	0.16
speed=slow,direction=down	speed=slow,direction=up	−0.13	−0.07	−0.01	0.01
speed=fast,direction=left	speed=slow,direction=left	−0.22	−0.16	−0.10	0.00
speed=fast,direction=left	speed=fast,direction=right	−0.05	0.01	0.07	1.00
speed=fast,direction=left	speed=slow,direction=right	−0.18	−0.11	−0.05	0.00
speed=fast,direction=left	speed=fast,direction=up	−0.10	−0.04	0.02	0.54
speed=fast,direction=left	speed=slow,direction=up	−0.23	−0.17	−0.10	0.00
speed=slow,direction=left	speed=fast,direction=right	0.11	0.17	0.23	0.00
speed=slow,direction=left	speed=slow,direction=right	−0.02	0.05	0.11	0.30
speed=slow,direction=left	speed=fast,direction=up	0.06	0.12	0.18	0.00
speed=slow,direction=left	speed=slow,direction=up	−0.07	−0.00	0.06	1.00
speed=fast,direction=right	speed=slow,direction=right	−0.19	−0.12	−0.06	0.00
speed=fast,direction=right	speed=fast,direction=up	−0.11	−0.05	0.01	0.18
speed=fast,direction=right	speed=slow,direction=up	−0.24	−0.18	−0.12	0.00
speed=slow,direction=right	speed=fast,direction=up	0.01	0.07	0.14	0.01
speed=slow,direction=right	speed=slow,direction=up	−0.11	−0.05	0.01	0.19
speed=fast,direction=up	speed=slow,direction=up	−0.19	−0.12	−0.06	0.00

Supplementary Table 7 | Group data analysis using a post-hoc Tukey's comparison for the interaction between speed and direction on reaction time.

Factor	df	SS	MS	EMS	F	p-value
subject	9	6.83	0.76	35.3032*V(subject)+5.88386*V(subject*speed, pert.)+V(Error)	13.17	4.09×10^{-12}
speed, pert.	5	11.98	2.40	128.801*Q(speed, pert.)+12.8801*V(subject*speed, pert.)+V(Error)	23.23	9.56×10^{-12}
RT	1	0.02	0.02	173.667*Q(RT)+17.3667*V(subject*RT)+28.9446*Q(speed, pert.*RT)+V(Error)	0.86	3.59×10^{-01}
subject*speed, pert.	45	5.32	0.12	15.2159*V(subject*speed, pert.)+V(Error)	6.12	1.03×10^{-30}
subject*RT	9	0.59	0.07	95.875*V(subject*RT)+V(Error)	3.41	3.72×10^{-04}
speed, pert.*RT	5	0.17	0.03	115.594*Q(speed, pert.*RT)+V(Error)	1.73	1.25×10^{-01}
Error	1235	23.89	0.02	V(Error)		
Total	1309	63.95				

Supplementary Table 8 | Group data analysis using a three-way ANOVA of main effects and interactions that subject, type of perturbation (speed, pert.), and reaction time (RT) have on speed error. (df = degrees of freedom, SS = Sum of Squares, MS = Mean Squares, EMS = Expected Mean Squares, F = F-statistic)

Factor A	Factor B	Lower Limit	Mean Difference	Upper Limit	p-value
subject=1	subject=2	−0.14	−0.05	0.05	0.85
subject=1	subject=3	−0.47	−0.37	−0.27	0.00
subject=1	subject=4	−0.34	−0.22	−0.11	0.00
subject=1	subject=5	−0.54	−0.45	−0.35	0.00
subject=1	subject=6	−0.34	−0.25	−0.15	0.00
subject=1	subject=7	−0.34	−0.24	−0.14	0.00
subject=1	subject=8	−0.37	−0.27	−0.16	0.00
subject=1	subject=9	−0.48	−0.36	−0.24	0.00
subject=1	subject=10	−0.40	−0.29	−0.17	0.00
subject=2	subject=3	−0.42	−0.33	−0.23	0.00
subject=2	subject=4	−0.28	−0.17	−0.06	0.00
subject=2	subject=5	−0.49	−0.40	−0.31	0.00
subject=2	subject=6	−0.29	−0.20	−0.11	0.00
subject=2	subject=7	−0.29	−0.20	−0.10	0.00
subject=2	subject=8	−0.32	−0.22	−0.12	0.00
subject=2	subject=9	−0.43	−0.31	−0.19	0.00
subject=2	subject=10	−0.35	−0.24	−0.13	0.00
subject=3	subject=4	0.03	0.15	0.27	0.00
subject=3	subject=5	−0.17	−0.07	0.03	0.36
subject=3	subject=6	0.03	0.13	0.23	0.00
subject=3	subject=7	0.03	0.13	0.23	0.00
subject=3	subject=8	−0.00	0.11	0.21	0.06
subject=3	subject=9	−0.11	0.01	0.13	1.00
subject=3	subject=10	−0.03	0.09	0.20	0.30
subject=4	subject=5	−0.34	−0.23	−0.11	0.00
subject=4	subject=6	−0.14	−0.02	0.09	1.00
subject=4	subject=7	−0.14	−0.02	0.09	1.00
subject=4	subject=8	−0.17	−0.05	0.08	0.98
subject=4	subject=9	−0.27	−0.14	−0.00	0.05
subject=4	subject=10	−0.19	−0.06	0.06	0.85
subject=5	subject=6	0.11	0.20	0.29	0.00
subject=5	subject=7	0.11	0.20	0.30	0.00
subject=5	subject=8	0.08	0.18	0.28	0.00
subject=5	subject=9	−0.03	0.09	0.21	0.38
subject=5	subject=10	0.05	0.16	0.27	0.00
subject=6	subject=7	−0.09	0.00	0.09	1.00
subject=6	subject=8	−0.12	−0.02	0.08	1.00
subject=6	subject=9	−0.23	−0.11	0.01	0.09
subject=6	subject=10	−0.15	−0.04	0.07	0.98
subject=7	subject=8	−0.12	−0.02	0.08	1.00
subject=7	subject=9	−0.23	−0.11	0.00	0.07
subject=7	subject=10	−0.15	−0.04	0.07	0.97
subject=8	subject=9	−0.22	−0.09	0.04	0.41
subject=8	subject=10	−0.13	−0.02	0.10	1.00
subject=9	subject=10	−0.06	0.07	0.21	0.77

Supplementary Table 9 | Group data analysis using a post-hoc Tukey's comparison for the main effect of subject on speed error.

Factor A	Factor B	Lower Limit	Mean Difference	Upper Limit	p-value
speed, pert.=fast, away	speed, pert.=fast, towards	0.02	0.10	0.19	0.01
speed, pert.=fast, away	speed, pert.=fast, unpert	0.09	0.14	0.19	0.00
speed, pert.=fast, away	speed, pert.=slow, away	0.17	0.24	0.31	0.00
speed, pert.=fast, away	speed, pert.=slow, towards	0.31	0.41	0.51	0.00
speed, pert.=fast, away	speed, pert.=slow, unpert	0.29	0.34	0.39	0.00
speed, pert.=fast, towards	speed, pert.=fast, unpert	-0.04	0.03	0.11	0.77
speed, pert.=fast, towards	speed, pert.=slow, away	0.05	0.13	0.22	0.00
speed, pert.=fast, towards	speed, pert.=slow, towards	0.19	0.30	0.42	0.00
speed, pert.=fast, towards	speed, pert.=slow, unpert	0.16	0.24	0.31	0.00
speed, pert.=fast, unpert	speed, pert.=slow, away	0.05	0.10	0.15	0.00
speed, pert.=fast, unpert	speed, pert.=slow, towards	0.18	0.27	0.36	0.00
speed, pert.=fast, unpert	speed, pert.=slow, unpert	0.17	0.20	0.23	0.00
speed, pert.=slow, away	speed, pert.=slow, towards	0.07	0.17	0.27	0.00
speed, pert.=slow, away	speed, pert.=slow, unpert	0.05	0.10	0.15	0.00
speed, pert.=slow, towards	speed, pert.=slow, unpert	-0.16	-0.07	0.02	0.28

Supplementary Table 10 | Group data analysis using a post-hoc Tukey's comparison for the main effect of type of perturbation on speed error.

Subject	Internal states		Reaction time									Speed error									
	α^{SE}	α^{P}	β^{0}_{RT}	$\beta^{\text{SE}}_{\text{RT}}$	$\beta^{\text{P}}_{\text{RT}}$	β^{fast}	β^{slow}	β^{down}	β^{right}	β^{up}	β^{left}	β^{0}_{SE}	$\beta^{\text{SE}}_{\text{SE}}$	$\beta^{\text{P}}_{\text{SE}}$	$\beta^{\text{RT}}_{\text{SE}}$	$\beta^{\text{fast, unpert.}}$	$\beta^{\text{fast,}}_{\text{towards}}$	$\beta^{\text{fast, away}}$	$\beta^{\text{slow, unpert.}}$	$\beta^{\text{slow,}}_{\text{towards}}$	$\beta^{\text{slow, away}}$
1	0.80	0.38	0.01	0	0.08	0.13	0	-0.22	-0.22	0.14	0	-0.01	0.03	0.02	-0.12	0.05	-0.28	0	-0.23	0.05	-0.30
2	0.99	0.98	-0.22	0.46	0.34	0.11	0	0.12	-0.03	0.53	0	-0.48	0.04	-0.01	0.13	0.28	-0.05	0.21	0	0.44	0.25
3	0.79	0.76	0.05	0.15	0.19	0.17	0	-0.33	-0.23	-0.01	0	-0.19	0.03	-0.01	0.17	0.20	-0.14	0.31	0	0.50	0.05
4	0.99	0.99	0.42	0.16	0.12	-0.19	0	-0.44	-0.33	0	-0.40	-0.63	-0.08	0.08	0.17	0.57	0.40	0.58	0	0.57	0.52
5	0.99	0.99	0.55	0.42	-0.34	-0.82	0	-0.42	-0.29	-0.02	0	0.45	-0.05	0.05	0.11	-0.31	-0.53	0	-0.39	-0.21	-0.47
6	0.99	0.94	-0.65	-0.27	-0.35	0	1.23	-0.13	0	0.02	0.19	0.18	-0.02	-0.03	0.13	-0.24	-0.38	-0.31	-0.66	0	-0.31
7	0.85	0.99	0.33	-0.14	0.50	-0.87	0	0.34	-0.30	0.10	0	-0.24	-0.03	0.01	0.20	0.03	0.03	0	-0.17	0.48	0.17
8	0.40	0.01	-0.67	-0.24	0.10	0	1.14	0	-0.12	0.47	0.33	-0.25	0.01	-0.01	0.27	0.12	-0.01	0	-0.02	0.22	0.02
9	0.99	0.78	-0.71	-0.40	-0.16	0	1.07	0.03	0.18	0.33	0	-0.40	-0.03	-0.01	0.22	0.39	0.21	0.63	0	0.51	0.26
10	0.98	0.99	0.89	-0.24	0.36	-1.48	0	-0.14	0.09	-0.56	0	0.02	-0.09	0.10	0.08	0.05	-0.06	0	-0.05	0	-0.03

Supplementary Table 11 | Weights of model for each subject used in equations (3) and (4) for α's and equations (1) and (2) for β's.
