# Supplementary material for: Internal states as a source of subject-dependent movement variability are represented by large-scale brain networks
Source: Nat Commun. 2023 Nov 29;14:7837. doi: 10.1038/s41467-023-43257-4 (PMC10687170; doi:10.1038/s41467-023-43257-4)
Supplement: Supplementary file 3 — Reporting Summary [file 41467_2023_43257_MOESM3_ESM.pdf]

nature portfolio

Corresponding author(s): Macauley Smith Breault

Last updated by author(s): Oct 18, 2023

Reporting Summary

Nature Portfolio wishes to improve the reproducibility of the work that we publish. This form provides structure for consistency and transparency in reporting. For further information on Nature Portfolio policies, see our Editorial Policies and the Editorial Policy Checklist.

Statistics

For all statistical analyses, confirm that the following items are present in the figure legend, table legend, main text, or Methods section.

n/a	Confirmed	
☐	☒	The exact sample size (*n*) for each experimental group/condition, given as a discrete number and unit of measurement
☐	☒	A statement on whether measurements were taken from distinct samples or whether the same sample was measured repeatedly
☐	☒	The statistical test(s) used AND whether they are one- or two-sided<br>*Only common tests should be described solely by name; describe more complex techniques in the Methods section.*
☐	☒	A description of all covariates tested
☐	☒	A description of any assumptions or corrections, such as tests of normality and adjustment for multiple comparisons
☐	☒	A full description of the statistical parameters including central tendency (e.g. means) or other basic estimates (e.g. regression coefficient) AND variation (e.g. standard deviation) or associated estimates of uncertainty (e.g. confidence intervals)
☐	☒	For null hypothesis testing, the test statistic (e.g. *F*, *t*, *r*) with confidence intervals, effect sizes, degrees of freedom and *P* value noted<br>*Give P values as exact values whenever suitable.*
☒	☐	For Bayesian analysis, information on the choice of priors and Markov chain Monte Carlo settings
☒	☐	For hierarchical and complex designs, identification of the appropriate level for tests and full reporting of outcomes
☐	☒	Estimates of effect sizes (e.g. Cohen's *d*, Pearson's *r*), indicating how they were calculated

Our web collection on statistics for biologists contains articles on many of the points above.

Software and code

Policy information about availability of computer code

Data collection	- Clinical electrophysiology acquiring system (Neurofax EEG-1200, Nihon Kohden, USA)<br>- InMotion2 robotic manipulandum (Interactive Motion Technologies, USA)<br>- MonkeyLogic 2.72 (MATLAB)
Data analysis	- Custom code for analysis (MATLAB 2021b)<br>- Freesurfer v.6.0.0 (python 3.6+)<br>- Fieldtrip 20191008 (MATLAB 2021b)

For manuscripts utilizing custom algorithms or software that are central to the research but not yet described in published literature, software must be made available to editors and reviewers. We strongly encourage code deposition in a community repository (e.g. GitHub). See the Nature Portfolio guidelines for submitting code & software for further information.

Data

Policy information about availability of data

All manuscripts must include a data availability statement. This statement should provide the following information, where applicable:

- Accession codes, unique identifiers, or web links for publicly available datasets
- A description of any restrictions on data availability
- For clinical datasets or third party data, please ensure that the statement adheres to our policy

The raw SEEG data are protected and are not available due to restrictions on data sharing from Cleveland Clinic. The processed data that support the findings of this study are available on Johns Hopkins Research Data Repository with the identifier doi:10.7281/T1/PIVKJ7. The data generated in this study are also provided in the Source Data file.

Research involving human participants, their data, or biological material

Policy information about studies with human participants or human data. See also policy information about sex, gender (identity/presentation), and sexual orientation and race, ethnicity and racism.

Reporting on sex and gender	Sex- and gender-based analyses were not performed and information regarding gender was not explicitly collected. Due to the initially small sample size used in this study, stratifying patients further into sex- or gender-based groups would greatly reduce the power and generalizability of our findings. More importantly, there is no prior evidence to suggest that significant differences exist between different sex- or gender-based groups regarding intracranial electrophysiology, therefore, we did not feel that performing such analyses would provide any additional or beneficial information. The sex of participants is reported in this study.
Reporting on race, ethnicity, or other socially relevant groupings	Race, ethnicity, or other socially relevant groupings were not explicitly collected for this study.
Population characteristics	Study consisted of ten human participants (seven females and three males; mean age of 34 years) who were implanted with intracranial SEEG depth electrodes for clinical treatment of their epilepsy to identify Epileptogenic Zone (EZ) for possible resection. See table 1 for clinically relevant information about the participants.
Recruitment	Participants were recruited after discussion with the clinical team concluded that each participant would be an appropriate candidate for this study. Criteria included that participants were over the age of 18, able to provide consent, and able to perform the study task. We do not believe that any notable biases exist within the selection criteria that would significantly affect the results of our study. Subjects were introduced to this study by a member of their clinical team 24 hours before they were approached by a member of the research staff to participate.
Ethics oversight	The study protocol, including experimental paradigms and collection of relevant clinical and demographic data, was approved by the Cleveland Clinic Institutional Review Board. Subject criteria required volunteering individuals to be over the age of 18 with the ability to provide informed consent and able to perform the motor task. A data-sharing agreement between the Cleveland Clinic and Johns Hopkins University was approved by the legal teams of both institutions.

Note that full information on the approval of the study protocol must also be provided in the manuscript.

Field-specific reporting

Please select the one below that is the best fit for your research. If you are not sure, read the appropriate sections before making your selection.

☐ Life sciences ☒ Behavioural & social sciences ☐ Ecological, evolutionary & environmental sciences

For a reference copy of the document with all sections, see nature.com/documents/nr-reporting-summary-flat.pdf

Behavioural & social sciences study design

All studies must disclose on these points even when the disclosure is negative.

Study description	Quantitative experimental
Research sample	Ten human participants (seven females and three males; mean age of 34 years) with medical refectory focal epilepsy. At this time, the only ethical method to record from the brain necessary for our study using SEEG depth electrodes in humans is while they are implanted for clinical purposes. The rationale for choosing this study sample was because, at this time, the only ethical method to record from the brain necessary for our study using SEEG depth electrodes in adult humans is while they are implanted for clinical purposes.
Sampling strategy	Convenience sampling. No sample-size calculation was performed. At this time, the only ethical method to record from the brain necessary for our study using SEEG depth electrodes in humans is while they are implanted for clinical purposes. As such, in vivo

	human data collection such as this are rare as they require close collaborations between clinicians and researchers. We sought to capture as many participants as we could within patient safety and compliance given the difficult experimental paradigm. Data saturation was determined after at least 10 unique subject were collected. Our sample-size is on par with other scientific studies that use SEEG. See: Huang, Yuhao, et al. (2021). The insulo-opercular cortex encodes food-specific content under controlled and naturalistic conditions. Nature Communications 12.1: 1-12.
Data collection	The SEEG electrophysiological data from the intracranial depth electrodes were collected in the Epilepsy Monitoring Unit (EMU) at the Cleveland Clinic using the clinical electrophysiology acquiring system (Neurofax EEG-1200, Nihon Kohden, USA). Electrophysiological data from the participants were simultaneously collected while they performed the motor task. Participants performed the motor task in the EMU using a behavioral control system, which consisted of a computer screen where visual task stimuli were shown, an InMotion2 robotic manipulandum (Interactive Motion Technologies, USA) which they used to interact with the task, and a Windows-based laptop computer which ran the motor task using a MATLAB-based software tool called MonkeyLogic. Participants used the robotic manipulandum to control the position of a cursor on the computer screen during the motor task restricted to a horizontal two-dimension plane relative to themselves. Each session consisted of participants performing as many trials as they could for 30 minutes. Only the researcher and a clinician were in the room with the participant during the experiment. All were blind to the hypothesis in this study at the time of data collection.
Timing	Dates of data collection (formatted by Month-Day-Year [Subject number]): 04-04-2013 [6], 07-15-2013 [8], 07-18-2013 [1], 08-29-2013 [9], 09-09-2013 [3], 09-10-2013 [4], 10-14-2013 [5], 01-23-2014 [10], 11-04-2014 [2], 05-26-2015 [7]. Gaps between collection periods were due to researcher and eligible participant availability. This study in no way impacts clinical workflow.
Data exclusions	Two additional participants (not presented here) were excluded after data collection. Both failed to perform the motor task for the entire 30 minute session. Additionally, three subjects (6,10,7) performed the motor task for two sessions on separate days. We chose to exclude their second session from analysis to avoid any possible learning effect on either brain and/or behavior.
Non-participation	Due to the focus on movement for our experimental design, participants whose EZ the clinical team hypothesized to be in the motor or premotor cortex, indicated by the presence of electrodes in said areas, were rejected from participating in the study.
Randomization	Participants were not allocated into experimental groups.

Reporting for specific materials, systems and methods

We require information from authors about some types of materials, experimental systems and methods used in many studies. Here, indicate whether each material, system or method listed is relevant to your study. If you are not sure if a list item applies to your research, read the appropriate section before selecting a response.

Materials & experimental systems

n/a	Involved in the study
☒	☐ Antibodies
☒	☐ Eukaryotic cell lines
☒	☐ Palaeontology and archaeology
☒	☐ Animals and other organisms
☒	☐ Clinical data
☒	☐ Dual use research of concern
☒	☐ Plants

Methods

n/a	Involved in the study
☒	☐ ChIP-seq
☒	☐ Flow cytometry
☐	☒ MRI-based neuroimaging

Plants

Seed stocks	*Report on the source of all seed stocks or other plant material used. If applicable, state the seed stock centre and catalogue number. If plant specimens were collected from the field, describe the collection location, date and sampling procedures.*
Novel plant genotypes	*Describe the methods by which all novel plant genotypes were produced. This includes those generated by transgenic approaches, gene editing, chemical/radiation-based mutagenesis and hybridization. For transgenic lines, describe the transformation method, the number of independent lines analyzed and the generation upon which experiments were performed. For gene-edited lines, describe the editor used, the endogenous sequence targeted for editing, the targeting guide RNA sequence (if applicable) and how the editor was applied.*
Authentication	*Describe any authentication procedures for each seed stock used or novel genotype generated. Describe any experiments used to assess the effect of a mutation and, where applicable, how potential secondary effects (e.g. second site T-DNA insertions, mosiacism, off-target gene editing) were examined.*

Magnetic resonance imaging

Experimental design

Design type	Non-functional preoperative MRI

Design specifications	Non-functional preoperative MRI
Behavioral performance measures	Non-functional preoperative MRI

Acquisition

Imaging type(s)	Structural
Field strength	1.5T or 3T
Sequence & imaging parameters	MPRAGE, slice thickness: 1-mm, repetition time (TR): 2200-ms, flip angle: 8, echo time: 2450-ms
Area of acquisition	Whole brain

Diffusion MRI ☐ Used ☒ Not used

Preprocessing

Preprocessing software	Freesurfer v.6.0.0 and Fieldtrip 20191008
Normalization	Non-linear spatial normalization algorithm contained within the Fieldtrip package. See: Ashburner, J., & Friston, K.J. (1999). Nonlinear spatial normalization using basis functions. Human Brain Mapping. 7: 254-266.
Normalization template	MN1152
Noise and artifact removal	Not applicable since non-functional
Volume censoring	Not applicable since non-functional

Statistical modeling & inference

Model type and settings	Non-parametric cluster statistic was used to find windows of spectral data that covaried across trials with internal states estimated from behavioral models for the population.
Effect(s) tested	Addition of internal states to behavioral model were supported by ANOVA tests showing that trial conditions (speed, direction, perturbation type) alone could not account for outputs of behavioral models (reaction time and speed error0.

Specify type of analysis: ☐ Whole brain ☒ ROI-based ☐ Both

Anatomical location(s)	Coordinates of each electrode in subject space were found using semi-automatic protocol. See: Stolk, Arjen, et al. (2018). Integrated analysis of anatomical and electrophysiological human intracranial data. Nature protocols. 13.7: 1699-1723.<br>Electrode coordinates in subject space were mapped to anatomical location using Destrieux atlas from cortical parcellation during Freesurfer in subject space, as outlined in Stolk 2018.<br>The anatomical location of each electrode from semi-automatic protocol were verified by clinicians before analysis.<br>Electrode coordinates in subject space were then mapped to template space (cvs_avg35_inMNI152) for visualization purposes only. See: Hamilton, Liberty S., et al. (2017). Semi-automated anatomical labeling and inter-subject warping of high-density intracranial recording electrodes in electrocorticography. Frontiers in Neuroinformatics. 11: 62.
Statistic type for inference<br>(See Eklund et al. 2016)	Windows of time-frequency in the neural activity of a ROI that covaried with internal states (from behavioral models) in the population were found using non-parametric cluster statistic (two-tailed permutation test, N=1000, = 0.05). See: Maris, Eric, and Robert Oostenveld. (2007). Nonparametric statistical testing of EEG-and MEG-data. Journal of neuroscience methods 164.1: 177-190.
Correction	Clusters that were too small (i.e., had windows less than 250 ms in time, one octave in frequency, or area smaller than the minimum time and frequency windows specified) were discarded. A FDR (q = 0.015) was applied to correct for multiple comparisons between regions and epochs.

Models & analysis

n/a	Involved in the study
☐	☒ Functional and/or effective connectivity
☒	☐ Graph analysis
☐	☒ Multivariate modeling or predictive analysis

Functional and/or effective connectivity	Functional connectivity between ROIs were calculated by taking the Pearson correlation between the average neural activity within each cluster per trial.
Multivariate modeling and predictive analysis	Behavioral models were constructed for each subject using state-space representation that estimated movement variables (reaction time and speed error) as a function of trial conditions and two variables called internal states. Internal states evolve based on the history of errors and perturbations. Model variables were found using methods for generalized linear model that optimized the maximum likelihood estimation and were trained on all trials.
